# Morbidity and Mortality: A Case Report of Metastatic Bone Disease

**DOI:** 10.7759/cureus.3781

**Published:** 2018-12-27

**Authors:** Muhammad Siddique Pir, Jumana Jaloudi, Mariam Mir, Najam Saqib, Douglas Klamp

**Affiliations:** 1 Department of Internal Medicine, The Wright Center for Graduate Medical Education, Scranton, USA

**Keywords:** prostate cancer, multiple myeloma, lytic bone lesions, screening, metastatic bone disease, hypercalcemia, prostate-specific antigen

## Abstract

Metastatic prostate cancer and multiple myeloma (MM) usually present with bone lesions, posing a diagnostic challenge in males presenting in late stages. In this case report, an 86-year-old male who had not seen a physician in over 30 years presented with complaints of hip pain and progressive difficulty in walking for three weeks. Outpatient X-ray of the right hip showed multiple lytic bone lesions, raising suspicion of MM. Other laboratory tests revealed elevated serum calcium and elevated prostate-specific antigen (PSA), supporting a diagnosis of prostate cancer. The patient was admitted for further workup. Magnetic resonance imaging (MRI) of the spine showed diffuse metastatic disease throughout the spine as well as pelvis with multilevel central canal and neuro-foraminal narrowing due to degenerative changes. Central canal narrowing at L1-L2 due to tumor involvement could not be ruled out on MRI.

Subsequently, urology was consulted and the patient was taken to the operating room for prostate biopsy and possible bilateral orchiectomy. Two intraoperative prostate biopsies were negative for malignancy but patient underwent bilateral orchiectomy due to high clinical suspicion for prostate cancer. Bone lesions in the pelvis were so extensive that orthopedic surgeons recommended complete non-weight bearing as the risk of fracture with weight bearing was thought to be very high. Eventually, laboratory workup for MM came out to be positive. Radiation oncologist recommended radiation therapy; however, at this point, the patient refused further intervention. He opted for palliative care. Consequently, a bone marrow biopsy could not be obtained for a definitive diagnosis of MM. The patient was eventually discharged to a nursing home for hospice care.

This case sheds light on the importance of preventative care in routine outpatient setting, which can often screen, identify, and detect malignancies at earlier stages. It also signifies the importance of an interdisciplinary approach and precise knowledge in differentiating and diagnosing such malignancies. In our patient’s case, his extensive bone disease precluded his ability to be weight bearing which is an uncommon finding only seen in extensive metastatic bone disease. A definitive diagnosis is warranted to guide appropriate management.

## Introduction

Metastatic prostate cancer and multiple myeloma (MM) both commonly lead to bone involvement. In advance stages, it may become challenging to make a clinical diagnosis due to overlapping features. This case report highlights such a case with several features in support of each diagnosis. This case also sheds light on the importance of regular primary care follow up visits for preventive care. Prostate-specific antigen (PSA)-based prostate cancer screening (even though somewhat controversial) may, in fact, have a very consequential impact in such cases.

## Case presentation

**Figure 1:**
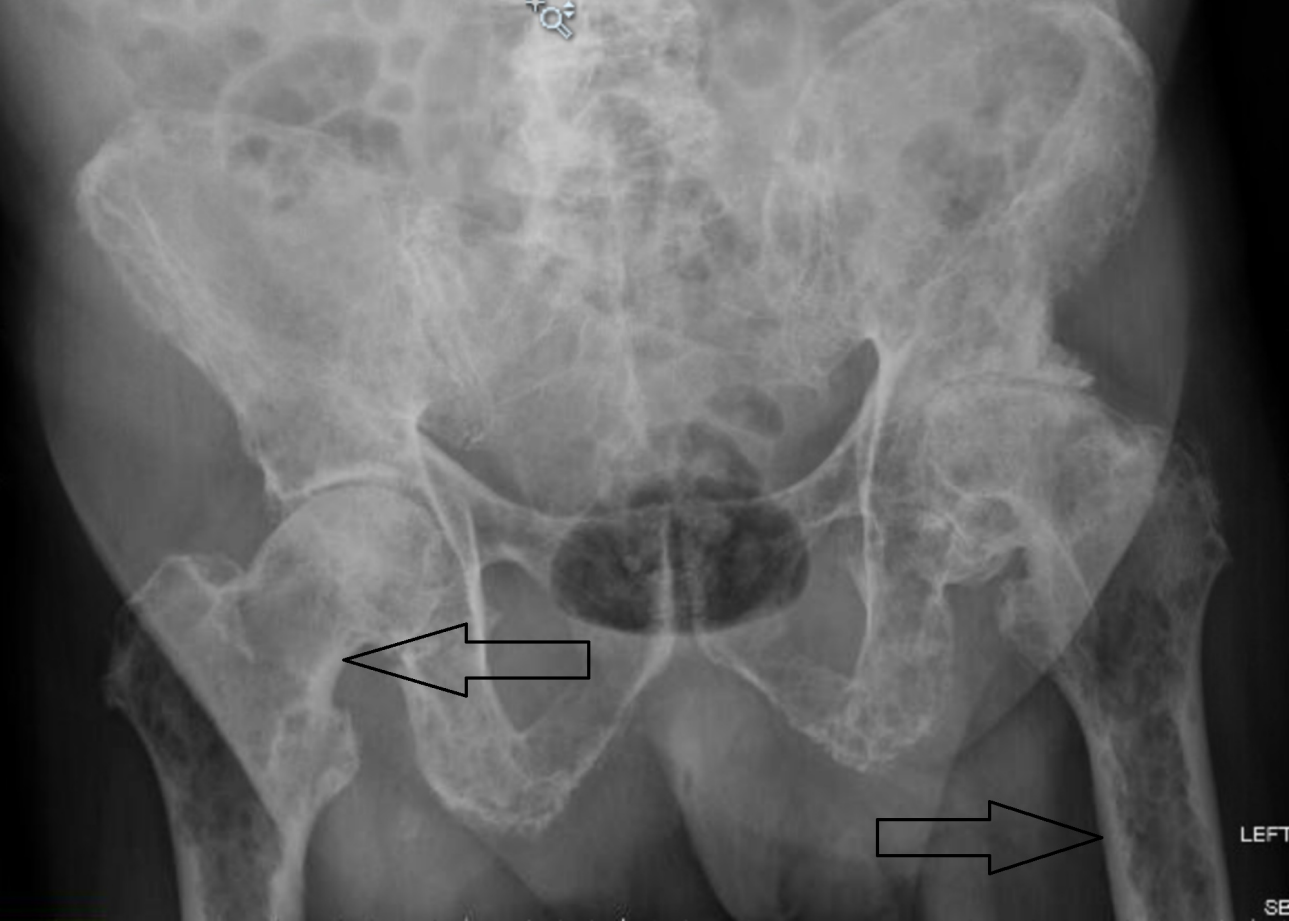
X-ray of the pelvis Showing extensive lytic bone lesions marked by arrows.

**Figure 2:**
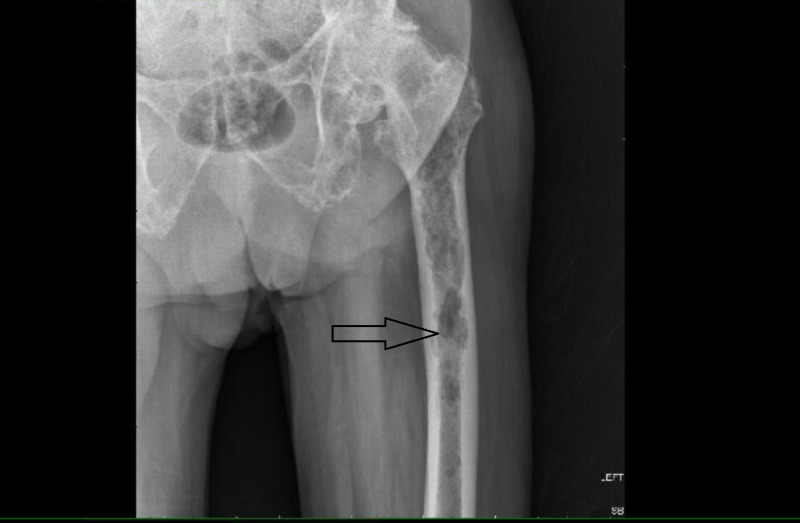
X-ray of the femur Showing multiple lytic lesions; arrow points towards one big lytic lesion.

**Figure 3:**
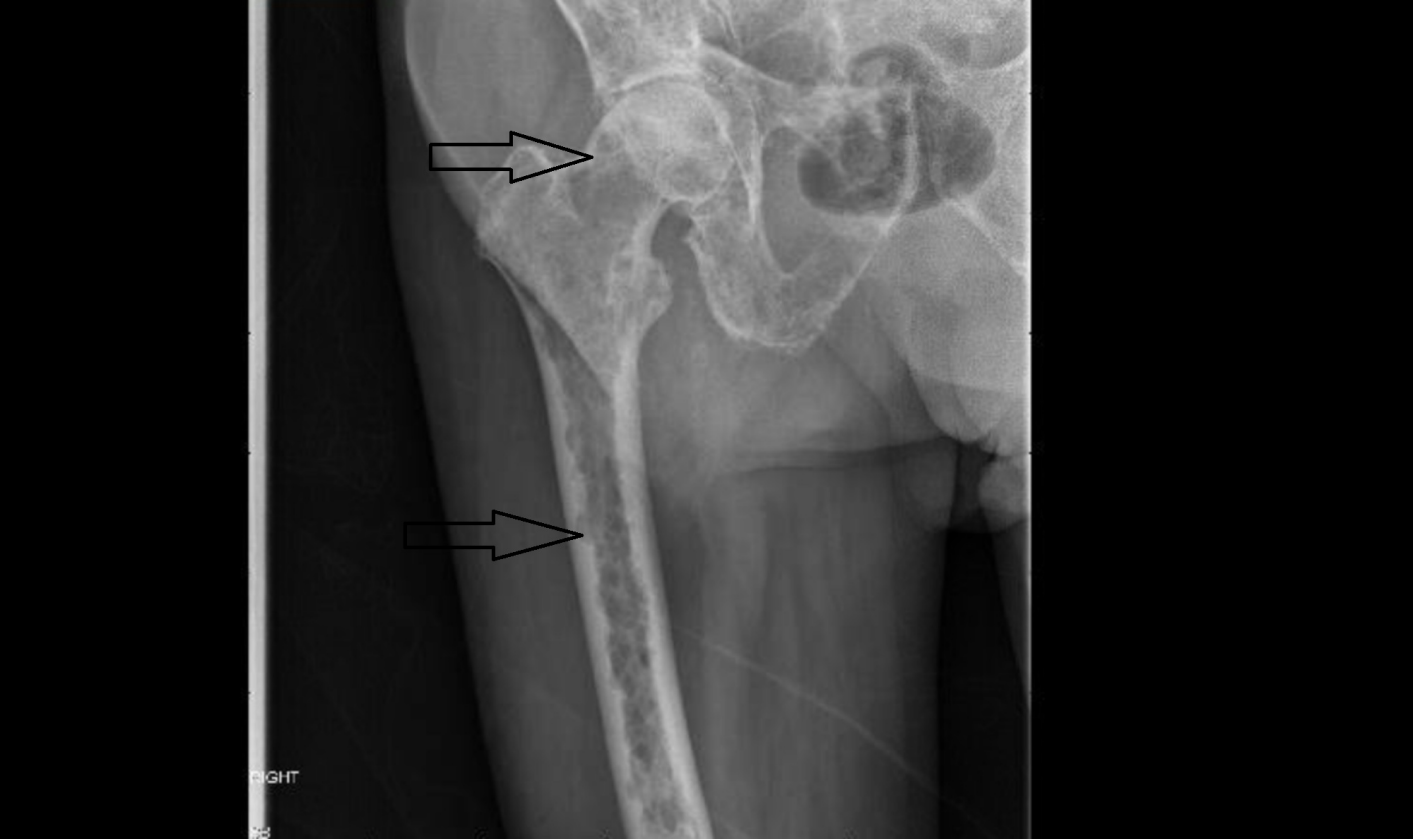
X-ray of the femur Showing multiple lytic lesions marked by arrows.

**Figure 4:**
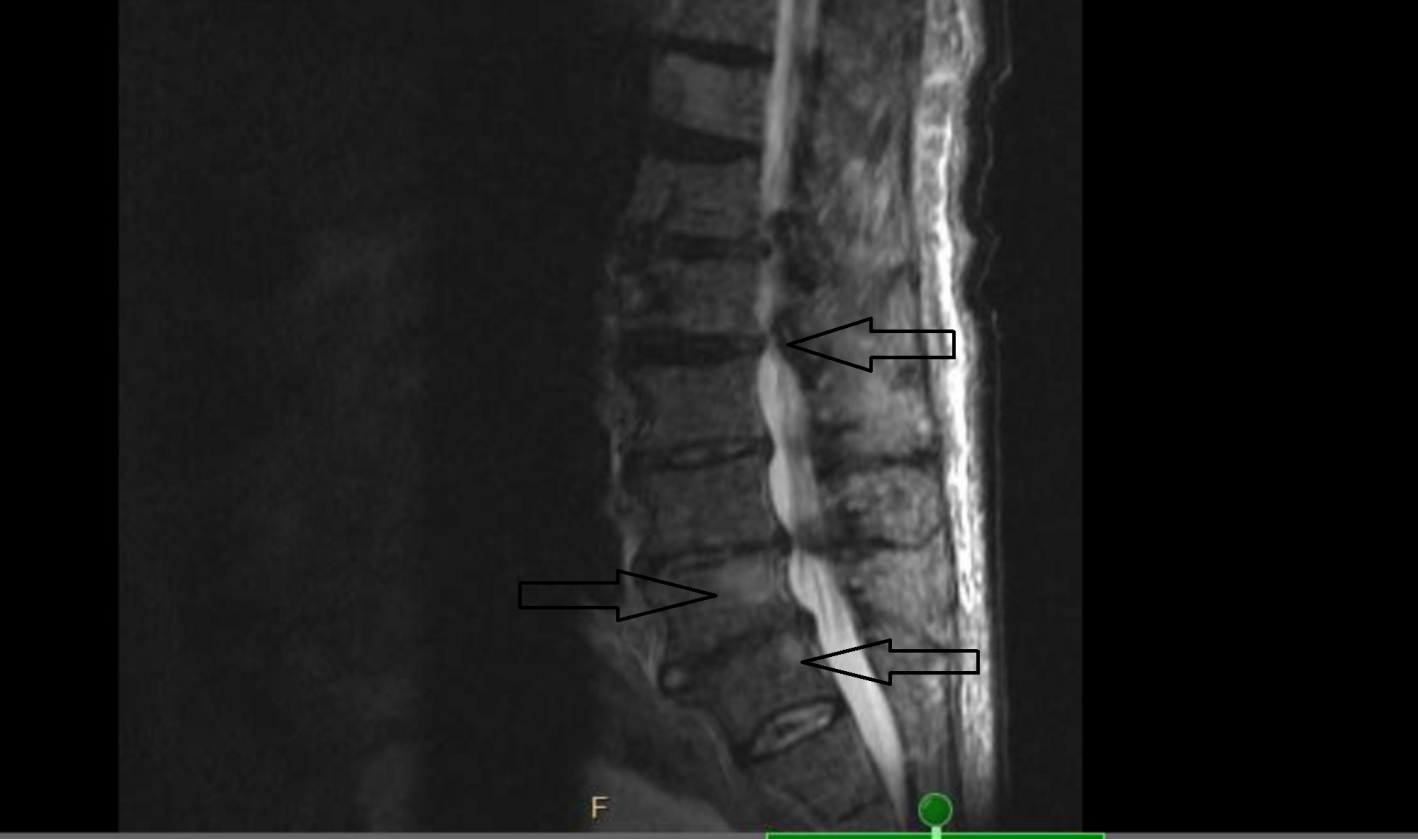
Magnetic resonance imaging (MRI) of the lumbar spine Showing indeterminate cord compression (marked by an arrow) and multiple bone lesions, two of them marked by arrows.

**Figure 5:**
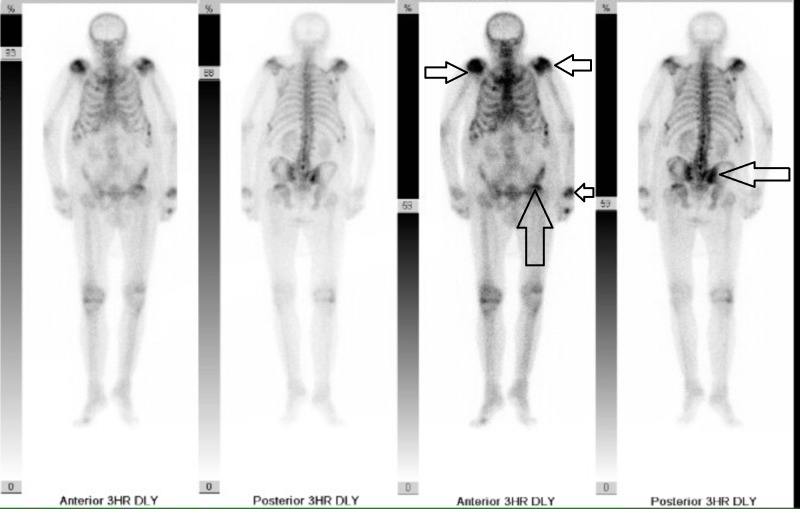
Bone scan Showing metastatic disease in multiple areas including the shoulders, left wrist, left side of the pelvis, ribs, and sternum.

An 86-year-old male with no known past medical history presented to his primary care physician with complaints of sudden onset of right hip pain and progressive difficulty in walking for three weeks. He did not follow-up with any physician for over 30 years. He reported severe pain that was exacerbated by any physical activity and movement. His pain was minimally relieved with rest and radiated to bilateral lower extremities. Outpatient chest and right hip X-rays revealed multiple lytic lesions, suggestive of metastatic disease. The patient was admitted to the hospital for further workup. X-rays of the pelvis revealed multiple lytic lesions throughout the entire pelvis and bilateral femurs (Figures 1-3). Laboratory tests were significant for the following: hemoglobin level of 9 g/dl, creatinine 2.8 mg/dl, calcium 13.5 mg/dl, albumin 2.7 g/dl, total serum protein 9.6 g/dl, globulin 7.1 g/dl, albumin to globulin ratio 0.3, free PSA 6.9ng/mL and total PSA 78.0 ng/mL. Urinalysis showed proteinuria and hematuria. Magnetic resonance imaging (MRI) of the spine revealed diffuse metastatic disease (Figure 4). A bone scan showed multiple foci of increased activity throughout bilateral ribs, spinous processes of the lumbar spine, bilateral shoulders, left iliac wing and left acetabulum consistent with metastatic disease (Figure 5). At this point, the working diagnoses included MM and metastatic prostate cancer. Urology proceeded with a prostate biopsy. Despite negative intra-operative frozen sections for prostate cancer, a bilateral orchiectomy was performed due to high clinical suspicion of prostate cancer. The aim of this procedure was to achieve early androgen deprivation to prevent spinal cord compression. The patient underwent the procedure without any complications. He was started on steroids for suspected spinal cord compression. Intravenous fluid hydration and zoledronic acid were administered for hypercalcemia. Subsequent laboratory tests showed improvement in calcium level. Serum creatinine level remained elevated between 2 mg/dl and 3 mg/dl. MM workup including serum protein electrophoresis, serum, and urine free kappa and lambda light chains were supportive of a diagnosis of MM: free kappa serum level 19.7 mg/dl, free lambda serum level 3099 mg/dl, free kappa urine 164 mg/L, free lambda urine 1500 mg/L, and free kappa/lambda urine ratio 0.11. Immunofixation was interpreted as monoclonal IgG lambda present. According to the Revised International Myeloma Working Group Diagnostic Criteria for MM, tissue evidence of >10% of bone or extramedullary plasmacytoma is necessary for a definitive diagnosis of MM. It was recommended by orthopedic surgeons that the patient be on bed rest indefinitely due to high fracture risk as a result of his bone frailty. The patient was given the option to proceed with bone marrow biopsy and appropriate treatment based on results. Radiation therapy to help with the pain was also recommended. After extensive discussion about all diagnostic, therapeutic and palliative options patient opted for comfort measures only. He did not want to pursue further diagnostic workup as his quality of life was expected to be severely diminished. The patient was eventually discharged to a nursing home for hospice care.

## Discussion

Advanced prostate cancer and MM are two very distinct malignancies with differing underlying pathogenesis and management; however, they share several overlapping clinical features, especially in late or advanced disease, which may pose a diagnostic challenge for clinicians [1-2]. MM is typically associated with lytic bone lesions while prostate cancer typically presents with osteoblastic lesions; however, prostate cancer may present as osteolytic bone lesions as well [3-4]. Moreover, prostate cancer is the most common malignancy in males, especially in the elderly, making it a likely diagnosis in our case at initial presentation.

Early androgen deprivation therapy with bilateral orchiectomy or with chemical castration is recommended for suspected metastatic prostate cancer [5] especially in case of suspected or actual spinal cord compression. A study published in the South African Journal of Surgery in 2014 suggested that the diagnosis of advanced prostate cancer may be established based on clinical features and elevated PSA alone without tissue biopsy and androgen deprivation therapy may be initiated [6]. Our patient presented with evidence suggestive of impending cord compression, therefore, early aggressive therapy was suggested for androgen-deprivation and surgical castration was thought to be the best option. In our case, the patient’s bone metastasis was discovered at a very late stage. It is reasonable to argue that this patient’s disease may have been detected earlier by screening process; however, as far as prostate cancer screening is concerned there are no unified guidelines. The U.S. Preventive Services Task Force (USPSTF) used to recommend against PSA–based screening for prostate cancer [7]. Very recently the USPSTF backtracked on previous recommendation and suggested the PSA based screening is an ‘individual decision’. On the other hand, the American Urological Association (AUA) panel recommends shared decision-making for men aged 55-69 years that are considering prostate cancer screening. The greatest benefit of screening appears to be in men ages 55-69 years. The USPSTF Guidelines, AUA, American College of Physicians (ACP), American Cancer Society (ACS) and the Canadian Task Force on the Periodic Health Examination all stress the importance of informed decision making. Unfortunately, our patient did not follow up with any physician precluding his opportunity to have those important discussions.

MM is a cytogenetically heterogeneous clonal plasma cell proliferative disorder [8] and is almost always preceded by an asymptomatic premalignant stage termed monoclonal gammopathy of undetermined significance (MGUS) [9]. The International Myeloma Working Group Criteria defines MM as clonal bone marrow plasma cells more than 10% or biopsy-proven extramedullary bony or plasmacytoma and either a myeloma defining event (evidence of end organ damage such as hypercalcemia, renal insufficiency, anemia and bone lesions, commonly abbreviated as CRAB) or any biomarkers of malignancy (clonal bone marrow plasma cell percentage more than 60%, uninvolved serum free light chain ratio >100 and more than one focal lesion on MRI studies). Clinical presentation of MM may overlap with that of advanced prostatic cancer with bony metastasis. In our case, the patient did fulfill the CRAB criteria, which is one of the diagnostic features of MM; however, we could not make a definitive diagnosis without a bone marrow biopsy.

## Conclusions

This case report sheds light on the importance of preventative care in routine outpatient care, which can often screen, identify, and detect malignancies at earlier stages. It also signifies the importance of an interdisciplinary approach and precise knowledge in differentiating and diagnosing such malignancies. In our patient’s case, his extensive bone disease precluded his ability to be weight bearing which is an uncommon finding only seen in extensive metastatic bone disease. A definitive diagnosis is warranted to guide appropriate therapy and management of all malignancies. This case also demonstrates the overlap between clinical features of MM and metastatic prostate cancer. Ultimately, patient preferences dictate the extent of diagnostic evaluation and management; therefore, it is of utmost importance to engage patients in their ongoing medical and preventative care.
